# Induced Pluripotent Stem Cells Derived from Alzheimer’s Disease Patients: The Promise, the Hope and the Path Ahead

**DOI:** 10.3390/jcm3041402

**Published:** 2014-12-12

**Authors:** Kristine Freude, Carlota Pires, Poul Hyttel, Vanessa Jane Hall

**Affiliations:** Department of Veterinary Clinical and Animal Sciences, Faculty of Health and Medical Sciences, University of Copenhagen, Gronnegaardsvej 7, Frederiksberg C DK-1870, Denmark; E-Mails: kkf@sund.ku.dk (K.F.); cp@sund.ku.dk (C.P.); poh@sund.ku.dk (P.H.)

**Keywords:** induced pluripotent stem cells, Alzheimer’s disease, disease modeling, human

## Abstract

The future hope of generated induced pluripotent stem cells (iPS cells) from Alzheimer’s disease patients is multifold. Firstly, they may help to uncover novel mechanisms of the disease, which could lead to the development of new and unprecedented drugs for patients and secondly, they could also be directly used for screening and testing of potential new compounds for drug discovery. In addition, in the case of familial known mutations, these cells could be targeted by use of advanced gene-editing techniques to correct the mutation and be used for future cell transplantation therapies. This review summarizes the work so far in regards to production and characterization of iPS cell lines from both sporadic and familial Alzheimer’s patients and from other iPS cell lines that may help to model the disease. It provides a detailed comparison between published reports and states the present hurdles we face with this new technology. The promise of new gene-editing techniques and accelerated aging models also aim to move this field further by providing better control cell lines for comparisons and potentially better phenotypes, respectively.

## 1. Introduction

Alzheimer’s disease (AD) is an incurable age-associated disorder characterized by progressive neurodegeneration and is the most common type of dementia, currently affecting 35.6 million people worldwide, which is a figure that is expected to triple by 2050 [1]. The majority of cases have a development of late-onset symptoms (after the age of ~65), which include personality/behaviour changes and memory deficits, hindering general, everyday activities [2]. This late-onset form is most often the sporadic form of AD (SAD), whereby increasing age is the greatest risk factor, but may also be associated with unknown environmental exposures, or a family history of AD. Mutations in the polymorphic apolipoprotein E (*APOE*) gene are known to increase the risk in developing late-onset AD and it is further believed that this complex disease involves other susceptibility genes and/or spontaneous mutations in unknown genes [3,4,5]. Genetic factors account for approximately 80% of the risk for AD, and genome-wide association studies (GWAS) have identified several candidate genes besides *APOE* that may be associated with late-onset disease, including *ABCA7*, *BIN1*, *CD33*, *CLU*, *CR1*, *CD2AP*, *EPHA1*, *MS4A6A-MS4A4E*, *PICALM*, *HLA-DRB5-DRB1*, *SORL1*, *FERMT2*, *CASS4*, *PTK2B*, amongst others [6,7,8,9,10]. However, these susceptibility loci explain only around half of the total genetic variance and extensive further analyses are still necessary to characterize these candidate genes and elucidate their association with AD risk. Less than 5% of AD patients manifest symptoms at an earlier stage (before the age of 65),* i.e*., familial AD (FAD), which is linked to genetic mutations in one of three genes, including, amyloid precursor protein (APP), presenilin1 (PSEN1) and presenilin 2 (PSEN2) [11]. PSEN1 accounts for the majority of FAD cases, whereas, PSEN2 and APP mutations are rarer and some FAD cases are not caused by mutations on any of these genes [12]. In this review, we provide an overview of the current status in the development of patient-specific induced pluripotent stem (iPS) cells derived from AD patients and how these cells may help sufferers of the disease, with respect to basic research findings, drug discovery and other treatments that may prospectively benefit the patients.

## 2. AD Pathology and Progression

The pathophysiology of the disease is not well understood and considering the prevalence and poor prognosis of AD, there has been a research priority in developing disease models for studying pathogenicity and to aid in development of therapeutic approaches. The difficulty in accessing brain samples from patients, along with the fact that only post-mortem brain analysis allows a definite AD diagnosis, makes iPS cells technology highly relevant in this context. That is, these cells, which are produced from directly reprogrammed AD patient somatic cells (e.g., dermal fibroblasts) into neuronal cells [13], will help us gain access to the disease in a dish, which would be much easier to study.

Two pathological hallmarks are known to occur in the patient’s brain, however, it remains unclear which of these appear first and/or is mainly responsible for the disease’s progress [11,14]. One hallmark is the development of senile neuritic plaques, composed of extracellular accumulation of Amyloid-β (Aβ). These are formed from the extracellular deposition of Aβ monomers, which aggregate as amyloid fibrils outside of the neurons. There is much evidence to support the Amyloid hypothesis, which suggests these plaques are largely responsible for extensive synaptic loss and neuronal death in the disease [15,16]. Tauopathy, the second hallmark, refers to intracellular neurofibrillary tangles (NFTs) of hyperphosphorylated cytoskeletal protein tau, which are known to destabilize axonal microtubules and lead to cell loss [17]. These tangles are also considered by many to be the leading cause of the disease and which is described as the tau hypothesis. Both the Amyloid hypothesis and the tau hypothesis remain leading contenders for the underlying cause of the disease.

Staging of AD progression based on cortical neurofibrillary changes and increased expression of abnormal tau on postmortem brains reveal that Stage I (asymptomatic) initiates first in the periallococortical transentorhinal region of the temporal mesocortex located on the medial surface of the rhinal or collateral sulcus [18]. Stage II (asymptomatic) is evident to have spread to the layer pre-α or layer II of the entorhinal region and even deeper into the transentorhinal region. In stage III, lesions have progressed into the hippocampus, the layers pre-α and pri-α of the deep entorhinal layers, the temporal mesocortex and the high order sensory association areas of the temporal neocortex. In stage IV, the Ammon’s horn, the insular cortex and the medial temporal gyrus become affected [18]. Stage V is characterized by progression of lesions into the superior temporal gyrus and slightly affecting the premotor and first order sensory association areas of the neocortex [18]. The peristriatic region and parastriate area of the occipital lobe are also affected. The final stage VI ultimately resulting in death, is characterized by progression to the parastriate area and Brodman area of the first order sensory association areas and primary areas of the neocortex [18].

As a consequence, varying neural cells are affected, including, glia and neurons, such as pyramidal neurons, interneurons and specific neurons such as basal forebrain cholinergic neurons (BFCNs) [18,19,20,21,22,23,24]. In addition, extensive inflammation, glycation defects, deficiencies in the cell cycle in primary neurons, oxidative stress and endoplasmic reticulum stress-induced apoptosis have also been implicated in the disease [25,26,27,28,29,30,31,32,33].

## 3. Requirement for Further Basic Research into the Disease

With the difficulties in obtaining patient brain samples and a lack of adequate animal models of the disease, AD research is considerably hampered. Since discovering genetic mutations within FAD, several transgenic animal models (mostly rodent) containing single mutations (in PSEN, APP and tau) have been made [34,35,36,37]. These models have explained, to some extent, the pathogenicity of soluble Aβ oligomers and the connection between amyloidopathy and tauopathy, but failed to recapitulate the complete pathology observed in humans. For example, the transgenic AD mouse model (the PDAPP mouse), which overexpresses human APP containing the Indiana mutation (V717F) [34], has senile plaques, age-related Aβ accumulation and synaptic loss, however fails to show the presence of NFTs. Hsiao and colleagues developed the most studied AD transgenic model (Tg2576 mice), which overexpresses the human APP transgene containing the Swedish mutation (K670N/M671L) [35]. These mice also show age-related Aβ deposition, an increased Aβ1-42/Aβ1-40 soluble ratio, plus senile plaques, however, fail to show any neuronal loss [38]. Several other transgenic models have since been generated [39,40,41,42] and Aβ deposits and cognitive decline were widely reported in these models, but not NFTs or neuronal loss. The crossing of lines or production of double, triple or multiple mutations appear to mimic AD pathology even better, including in some cases, NFT-like lesions and neuronal death [43,44,45,46,47]. Unfortunately, the use of this multiple gene-strategy to induce widespread pathological features in the rodent differs considerably to familial human AD patients, which carry only single mutations. Furthermore, the use of these rodent models for pharmacological testing and evaluation of candidate drug targets has not led to the development of many successful drugs to date [48,49]. Staggeringly, it has been reported that hundreds of candidate drugs have failed during drug development [50] and it may simply be that our animal models are currently not optimal for either drug discovery or drug testing [49]. Emerging research indicates that* in vitro* human cell models of the disease may serve as more suitable models for recapitulating both the amyloid and tau hallmarks of the disease. One recent paper has reported that human neural progenitors cultured* in vitro* in 3D overexpressing either or both human *APP* and *PSEN1* genes containing *FAD* mutations could display both increased Aβ40 and Aβ42 expression, increased extracellular Aβ deposits, increased insoluble Aβ and increased phosphorylated tau (*p*-tau) in a proportion of differentiated neurons. Such evidence definitely helps to pave the way for future research into disease modeling using human-based cell culture systems.

Currently, there is no cure for AD or available drugs for the disease that can prevent progression long-term. Healthcare systems are over-loaded with dementia patients, which costs the society globally, around $604 billion (US dollars, 2010), making the disease a heavy economic burden on society [1]. With the ever-increasing age of the population and lack of highly successful clinical trials, it has become an urgent necessity to find enhanced treatments for AD. The prospect of even a slight improvement and delay in clinical onset of the disease within patients would have a great economic and social impact [51]. Reliable biomarkers are also very much needed since they allow for the* in vivo* detection of AD pathology in “normal” asymptomatic individuals [51]. Imaging technologies (*i.e.*, PET scans) and cerebrospinal fluid biomarkers have been developed and can detect certain indications of AD pathology in humans, but their predictive capacity at an individual level is still not reliable [11]. Moreover, these tests have been mainly used in a research environment and the question of whether they should be applied widely in clinics is still debatable, due to the lack of adequate testing in preclinical and clinical trials [11].

## 4. Hope in Modeling Alzheimer’s Disease Using Patient-Specific Induced Pluripotent Stem Cells

The development of iPS cells emerged in 2006, when mouse fibroblasts were successfully reprogrammed into pluripotent stem cells by retroviral-delivery of four transcription factors (Oct3/4, Sox2, c-Myc and Klf4), which activated an endogenous pluripotent state in somatic cells [52]. These iPS cells resembled embryonic stem cells (ESCs) [53], both in their expression profile, their ability to grow indefinitely and their ability to differentiate into all cell lineages of the body, including ectoderm, mesoderm and endoderm [52]. These cells have the potential to contribute to chimeras and to be transmitted through these chimeras into the germline, further proving their pluripotency [54]. The same and similar sets of factors were then applied to adult human fibroblasts [55,56,57]. It is reported these cells truthfully mimic human ESCs, however the reprogramming process is thought to induce some disparity at both the genetic and epigenetic levels [58,59,60,61]. Viral-delivery methods have shown good efficiency but also result in random integration of the transgenes into the genome, potentially leading to insertional mutagenesis and tumorigenicity, therefore restricting its use for future potential clinical trials [62]. More recently, integration-free reprogramming systems, including episomal plasmids containing the reprogramming factors, Sendai virus, direct mRNA, protein and small molecules, have all been successfully used for generation of potentially transgene-free human iPS cells [56,63,64,65]. Moreover, xeno- and feeder-free culture methods have also helped to decrease variability between lines generated [66,67]. In addition, other cell types from patients have been successfully and safely used for reprogramming. As an alternate to dermal skin fibroblasts (acquired from surgical skin biopsies), peripheral blood mononuclear cells (PBMCs) (which are easy to harvest from routine peripheral blood samples) have been used to isolate T cells and generate human iPS cells [68,69]. One advantage is that the reprogramming of these cells can also be done quickly, with no need for prior expansion. These protocols therefore facilitate the production of human iPS cells from human somatic cells with minimal invasiveness. Together, these mentioned advances in production of human iPS cells might allow research to fulfill the restricted guidelines of Good Manufacturing Practices (GMPs) for the development of clinically-approved iPS cells required in the regenerative medicine field [70].

## 5. Therapeutic Benefits

The discovery of iPS cells is groundbreaking, as it means that patient-specific cell lines can be established easily. Contrary to human iPS cells, human ESCs have been surrounded by ethical controversy due to the use of human embryos, which is a serious problem in terms of sample availability and public acceptance [71]. Human iPS cells are also clinically advantageous since the use of autologous tissue ideally surpasses the patient’s immune rejection, contrary to the allogeneic barriers of human ESCs [72]. Therapeutic cloning also allows the generation of pluripotent stem cells that are genetically similar to patients, however this requires the destruction of donor eggs or embryos and still has several technical issues [73,74]. Moreover, considering the unavailability of* in vitro* human disease models, human iPS cells could help to provide large numbers of patient-specific neuronal cells for research and clinical objectives. Pairing of both human iPS cell technology and advances in genome-editing technologies may also provide more robust findings since isogenic cell lines could lead to the replacement of age- and sex-matched controls [75,76,77,78]. Experimentally, this would allow for more phenotypic findings attributed to the genetic difference causing the disease, which would not be influenced by individual epigenetic differences [79]. Moreover, disease and population heterogeneity can also be diminished due to singular-patient origin of human iPS cells.

Regenerative medicine, including testing of transplantation of cells into live tissues and organs is ongoing for AD models of rodents, such as neural progenitor cells (NPCs) [80,81,82,83,84,85,86] and mesenchymal stem cells [87,88,89], but remains restricted in relation to transplantation of ESC or iPS-derived neural cells [80,90]. Some research, however, does suggest that implanted cells do not survive and that the beneficial effect may likely come from their secretion of BDNF (brain-derived neurotrophic factor) and GDNF (glial cell-derived neurotrophic factor) [82]. Due to improved immunocompatibility in the use of autologous iPS cells, there is considerable hope that differentiated progeny of patient-specific iPS cells may be favorable for transplantation.

**Figure 1:**
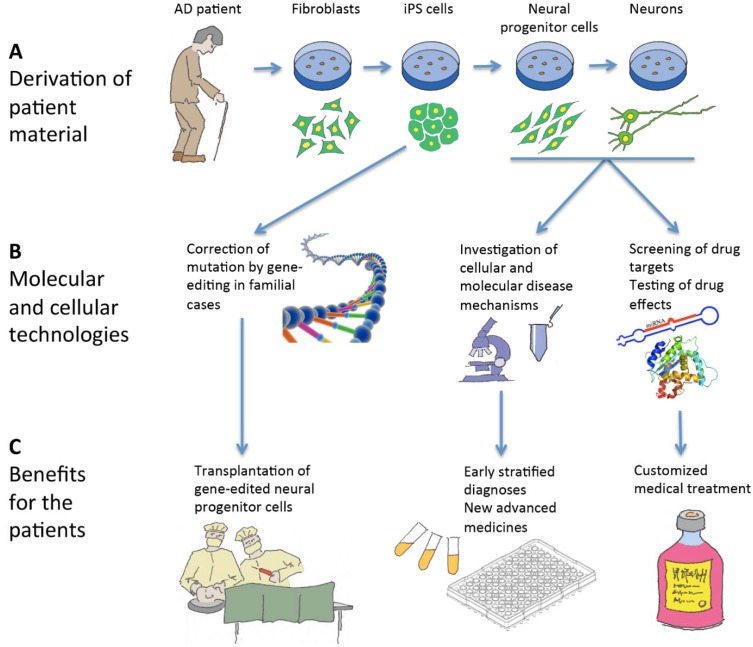
Schematic illustration of the use of induced pluripotent stem (iPS) cells in relation to Alzheimer’s disease (AD). (**A**) iPS cells are derived from a skin biopsy from an AD patient and differentiated into neural progenitor cells and neurons; (**B**) In familial cases, the disease-causing mutation can be corrected by gene-editing of the iPS cells, and neural progenitor cells and neurons can be used for research and drug screening; (**C**) The patients can in the long run benefit from these activities by cell therapy, better diagnostic procedures, customized treatments and novel medical approaches.

In addition, human iPS cells are already being used for drug development and screening in various diseases [91] to identify new and superior targets relevant for production of new drugs. In the future, it may even be possible to provide patient-customized cell screens from the iPS-derived cells to screen a panel of drugs in order to identify the most beneficial treatment plan for each individual patient [92]. This could have significant impact in treating this disease where patient variability is wide in response to certain drugs [93]. The development of patient-specific iPS cells may also help researchers to identify new mechanisms/biomarkers which may help lead to earlier diagnoses of the disease [94] as it is possible to culture early neurons or NPCs which may have underlying deficits related to the disease. It is also believed that earlier intervention is a key factor for a successful therapeutic strategy and an earlier diagnosis would be of extreme benefit to patients, as the initial stages of the disease could be treated whilst the patients are still early symptomatic [51]. It is crucial for clinical trials to target these early symptomatic patients, therefore facilitating therapeutic procedures to succeed in delaying, stopping or even preventing the cognitive decline [51]. We summarize the implications patient-specific iPS cells have on basic research as well as therapeutic benefits for AD in Figure 1.

## 6. Induced Pluripotent Stem Cells and Neural Cell Derivatives Have Been Produced from Several AD Patients

The discovery of iPS cells paved the way to model diseases by using patient-specific cells which can then be differentiated into disease relevant cell types. However, despite this breakthrough, there have been surprisingly only a handful of studies published on Alzheimer’s disease [95,96,97,98,99,100,101,102,103]. Induced pluripotent stem cells have now been derived from both familial and sporadic patients and these iPS cells have been differentiated into varying neurons and glia, which have been studied in respect to specific AD pathology. Here, we summarize the types of cells analyzed and the extent of their characterization (Table 1). The genetic backgrounds of patients that have been used to date, include, duplication or mutations of *APP*, mutations of *PSEN1* and *PSEN2*, in the case of familial AD [96,97,98,99,101,102] and mutations in APOE3/E4 resulting in both early sporadic and late sporadic forms of the disease [95]. It should be stated that in the case of one study [98], the *APP* (E693∆) mutation background (which is responsible for Alzheimer-type dementia [104]) showed no evident tau pathology and lack of fibrilization of Aβ peptides. Therefore not all hallmark pathologies would be anticipated in the iPSC-derived neurons. We also include a study where a Down-syndrome iPS cell line was used to model features of AD [100], since it could clearly model varying pathological features of the disease.

In each article, different types of neural cells have been analyzed, which have been derived using various differentiation protocols (see overview of protocols in Table 2). Cortical neurons have been studied by both Shi and colleagues and Kondo and colleagues [98,100]. Forebrain neurons have been studied by Muratore and colleagues [103]. Astrocytes (GFAP+) have also been studied by Kondo and colleagues [98] and basal forebrain cholinergic neurons (BFCNs) have been studied by Duan and colleagues [95]. GABAergic neurons have been studied by Koch and colleagues [97], whereas, more less-defined neurons (βIIITubulin+/MAP2+) have been studied by Israel and colleagues, Yagi and colleagues, Liu and colleagues and Sproul and colleagues [96,99,101,102]. Whilst some protocols used FACS to sort and purify the neural cell populations (e.g., sorting of CD24+CD184−CD44− neurons [96,101] and Lhx8+/Gbx1+ neurons [95]), it is without doubt that all the final cell populations analyzed had some degree of cell heterogeneity, as often observed by the percentages of positive cells stated. Three particular articles performed extensive characterization of the types of neurons generated. In the case of the generation of BFCNs, immunocytochemistry confirmed their mature features, since a large proportion expressed ChAT and VaChT, and all were found to be HB9 negative (a selective marker of motor neurons in the vertebrates) (Table 2).

**Table 1:** Phenotypes of neural cells analyzed from differentiated patient-specific induced pluripotent stem cells for studying Alzheimer’s disease.

Mutation	Cell Type Analyzed	Analyses Performed	Phenotype	Reference
Familial PSEN1 (A246E) 2 clones	Neurons (βIIITubulin+ MAP2+)	Extracellular Aβ Tau accumulation (HT7 antibody) Tangle formation Treatment with γ-secretase inhibitor and modulator of γ-secretase-mediated APP cleavage	Increased Aβ42:Aβ40 No Tau accumulation No tangle formation Decreased Aβ40+Aβ42 with γ-secretase inhibitor and modulator of APP cleavage	[99]
Familial PSEN2 (N141I) 2 clones				
Familial APP dup’n APP^Dp^1 3 clones	Neurons (βIII-Tubulin+ MAP2+) >90%	Genome-wide mRNA seq. Extracellular Aβ *p*-tau (Thr231) GSK-3β activity Treatment with γ-secretase and β-secretase inhibitors Endosome markers Synaptic markers	Increased Aβ40, Increased *p*-tau (Thr231), Increased aGSK-3β γ-/β-secretase inhibitors reduced Aβ40	[96]
Familial APP dup’n APP^Dp^2 3 clones			Increased Aβ40 Increased *p*-tau (Thr231), Increased aGSK-3β γ-/β-secretase inhibitors reduced Aβ40 β-secretase inhibitor reduced aGSK-3β+*p*-tau had large/very large Rab5 + early endosomes No change in synapsin I + puncta on dendrites	
Sporadic sAD1 3 clones			No change in Aβ40, No increase of *p*-tau (Thr231), No increase aGSK-3β γ-/β-secretase inhibitors reduced Ab40	
Sporadic sAD2 3 clones			No change in Aβ40 Increased *p*-tau (Thr^231^) and aGSK-3β γ-/β-secretase inhibitors reduced Aβ40 β-secretase inhibitor reduced aGSK-3β+*p*-tau had large/very large Rab5 + early endosomes No change in synapsin I + puncta on dendrites	
Familial PSEN1 (D385N)	It-NES progenitor cells (NESTIN+SOX2+)	Expression APP+ γ-secretase components Extracellular Aβ Aβ length qPCR	Dominant-negative effect on S3 cleavage of Notch in progenitors, decreased HES5	[97]
	Neurons (βIII-Tubulin+MAP2ab+ GABA+) + <10% Astrocyte (GFAP+)		Increased full-length APP Decreased Aβ40	
Familial PSEN1 (L166P)	It-NES cells (NESTIN+SOX2+)		Dominant-negative effect on S3 cleavage of Notch in progenitors, decreased HES5	
	Neurons (βIII-Tubulin+MAP2ab+ GABA+) <10% Astrocyte (GFAP+)		Decreased Aβ40	
Trisomy 21 DS1-iPS4	Cortical neurons; Early born, (TBR1+ βIII-Tubulin+ /CTIP2+ βIII-Tubulin+) 30% Late born, (BM2+ βIII-Tubulin+/SATB2+ βIII-Tubulin+) 20%–25% Functional synapses Glutamatergic (PSD95+)	Extracellular Aβ Aggregation of Aβ Treatment with γ-secretase inhibitor *p*-tau expression Cell death	Increased Aβ40 Increased Aβ42 (>70 days cultures) Increased Aβ42:Aβ40 Intracellular and extracellular Aβ42 aggregates Decreased Aβ40+Aβ42 with γ-secretase inhibitor *p*-tau localized in cell bodies and dendrites Increased secretion of total tau and *p*-tau Increased cell death (2 fold)	[100]
Familial APP(E693Δ) 3 clones	Cortical neurons (SATB2+TBR1+)	Extracellular Aβ Intracellular Aβ Aβ Oligomers Gene expression profiling ROS expression Aβ Oligomers ROS expression	Decreased Aβ40 and Aβ42 Elevated Aβ oligomers in neural cells Elevated levels of oxidative stress-related genes Elevated ROS	[98]
	Astrocytes		Elevated Aβ oligomers Elevated ROS	
Familial APP(V717L) 2 clones	Cortical neurons (SATB2+TBR1+)		Increased Aβ42, increased Aβ42:Aβ40 Elevated levels of oxidative stress-related genes	
Sporadic AD3E211 1 clone	Cortical neurons (SATB2+TBR1+)		No change in Aβ40 or Aβ42 Elevated levels of oxidative stress-related genes	
Sporadic AD8K213 1 clone	Cortical neurons (SATB2+TBR1+)		No change in Aβ40 or Aβ42 Elevated Aβ oligomers in neural cells Elevated levels of oxidative stress-related genes and ROS	
	Astrocytes		Elevated Aβ oligomers, Elevated ROS	
Sporadic Early ApoE3/E4 AG04402 (2 clones)	Basal forebrain cholinergic neurons (MAP2+ChAT+ VaChT+P75R+NKX2.1+HB9−) Expressed tetrodotoxin-sensitive voltage-activated currents and voltage-gated calcium channels	Extracellular Aβ Treatment with γ-secretase inhibitors Treatment with ionomycin + glutamate Fura-2 calcium imaging	Elevated Aβ42, Increased Aβ40 with γ-secretase inhibitor Increased susceptibility to glutamate-induced excitotoxic death Increased calcium transient	[95]
Sporadic Early, APOE3/E4 AG11414			Elevated Aβ42 Increased Aβ40 with γ-secretase inhibitor Susceptibility to cell death following calcium influx	
Sporadic Late APOE3/E4 AG05810			No elevated Aβ42 Increased susceptibility to glutamate-induced excitotoxic death Increased calcium transient	
Familial AG07872			Elevated Aβ42 Reduced Ab40 with γ-secretase inhibitor	
Familial PSEN1 (A246E) AG066848			No elevated Aβ42 Reduced Aβ40 with γ-secretase inhibitor	
Familial APP (V717I) (fAD1) (2 clones)	Forebrain neurons (MAP2+Tau+ βIII-Tubulin+Cux1+ TBR1+PSD95+ VGLUT1+)	Extracellular Aβ APP cleavage product expression Treatment with γ-secretase inhibitor Expression of tau Treatment with Aβ antibodies	APP holoprotein 1.4× increased Increased Aβ42:Aβ40 Increased Aβ42 Increased Aβ38 Decreased APPsα:APPsβ (Increased APPsβ) γ-secretase inhibitor blocked APPsβ cleavage Increased total tau Increased *p*-tau (Ser^262^) d100 Aβ antibodies blocked increased total tau (early differentiated neurons only)	[103]
(fAD2) asymptomatic (2 clones)				
Familial PSEN1 (A246E) (2 patients)	Neurons (βIII-Tubulin+MAP2+)	Extracellular Aβ Treatment with γ-secretase inhibitors	Increased Aβ42:Aβ40 Increased Aβ42 γ-secretase inhibitor lowered total Aβ, Aβ40, Aβ42, Aβ38	[101]
PSEN1 (H163R) asymptomatic			Increased Aβ42:Aβ40 Increased Aβ42 γ-secretase inhibitor lowered Aβ42	
PSEN1 (M146L)			Increased Aβ42:Aβ40 Increased Aβ42 γ-secretase inhibitor lowered Aβ42	
Familial; PSEN1 (A246E) (2 clones) 7671C/7768C	D14 immature neurons (79% NESTIN+ small pop’n TUJ1+)	Extracellular Aβ Total Aβ	Increased Aβ42:Aβ40 Increased *NLRP2*, *ASB9*, *NDP*	[102]
	Neurons Electrical signaling properties		Increased Aβ42:Aβ40	
PSEN1 (M146L) (2 clones) 8446B/8446D	D14 immature neurons (79% NESTIN+ small pop’n TUJ1+)		Increased Aβ42:Aβ40 Increased *NLRP2*, *ASB9*, *NDP*	
	Neurons Electrical signaling properties		Increased Aβ42:Aβ40	

**Table 2:** Reprogramming and differentiation strategies for induction of induced pluripotent stem cells and their neural progeny.

Reprogramming Strategy	Differentiation Protocol	Cell Type Formed	Reference
Retrovirus *OCT4*, *SOX2*, *KLF4*, *LIN28*, *NANOG* Human dermal fibroblasts	EB induction w/o bFGF 8 days EBs plated gelatin w/o bFGF 8 days Neuron induction w/o growth factors 2 weeks Added compound E or compound W 48 h	Neurons βIII-Tubulin+MAP2+	[99]
Retrovirus *OCT4*, *SOX2*, *KLF4*, *cMYC* Human dermal fibroblasts	Neuronal rosette induction on PA6 stromal cells 11 days NPCs isolated by FACS CD184+CD15+CD44−CD271− NPC cultured 4 weeks Neuron induction-BDNF/GDNF/cAMP 3 weeks CD24+CD184−CD44− neurons selected by FACS Cultured in BDNF/GDNF/cAMP 5 days	Neurons βIII-Tubulin+MAP2+ >90% VGluT1+ 15% GABA+ 8% Expressed tetrodotoxin-sensitive voltage-activated currents GABA+AMPA receptors Spontaneous inhibitory/excitatory synaptic currents	[96]
hESC (I3) transduced with lentivirus containing mutations in PS1 iPSC—Retrovirus Human dermal fibroblasts (PKa)	It-NES induction with bFGF+EGF+B27 Neuron induction—Matrigel w/o factors, +N2+B27+cAMP 4 weeks	Neurons βIII-Tubulin+ 80% Astrocytes 6%	[97]
Trisomy 21 Retrovirus *OCT4*, *SOX2*, *KLF4*, *cMYC* Human dermal fibroblasts	Matrigel+N2+B27+Noggin+SB431542 Dissociated and cultured with 3N+bFGF 100 days	Cortical neurons; Early born, TBR1+βIII-Tubulin+ CTIP2+βIII-Tubulin+ 30% Late born, BM2+βIII-Tubulin+ SATB2+βIII-Tubulin+ 20%–25% Functional synapses Glutamatergic+ PSD95+	[100]
Episomal vectors *SOX2*, *KLF4*, *OCT4*, *L-MYC*, *LIN28*, shRNA p53 Human dermal fibroblasts	EB induction DMEM/HamsF12+ 5% KSR+SB431542 8 days Neural induction—plated on Matrigel+N2+SB431542 16 days Cortical neuron induction—dissociated and cultured in NB media+B27+BDNF+GDNF+NT3 48 days As above, but on day 58 cortical neuron induction, cells passaged Repeated passages on day 96, 126, 156, 176	Cortical neurons SATB2+TBR1+ Astrocytes	[98]
Retroviral vector *Klf4*,* Oct4*, *Sox2*, *cMyc* Human fibroblasts	RA+bFGF 7 days Neurosphere formation w/o bFGF 7 days Neurospheres cultured with bFGF+EGF 4 days Neurospheres cultured with SHH+FGF8 3 days Dissociated and transfected with Lhx8/Gbx1-IRES-EGFP 2 days Lhx8+/Gbx1+ cells selected by FACS and cultured in NB media+ bFGF+NGF 2 weeks (+arabinoside from day 5–10 of NB culture step)	Basal forebrain cholinergic neurons 95% MAP2 66% ChAT VaChT+P75R+, NKX2.1+HB9− Expessed tetrodotoxin-sensitive voltage-activated currents, voltage-gated calcium channels	[95]
Lentivirus *OCT4*,* SOX2*, *cMYC*, *KLF4* Human dermal fibroblasts	Aggregates with iPS cell media 4 days + neural media+N2 2 days Aggregates plated on matrigel, Neural media + N2 10 days Suspension culture, neural media+B27+N2+cAMP+IGF1 7 days Neural rosettes selected manually or Neural Rosette selection agent Dissociated+plated on Matrigel+NBmedia+N2+B27+ cAMP+BDNF+GDNF+IGF1 35 days–76 days	Neurons 90% MAP2 Tau+, βIII-Tubulin+Cux1+Tbr1+PSD95+VGLUT1+ Spontaneous activity from microelectrode array	[103]
MMLV retrovirus *Oct4*, *Sox2*, *Klf4*, *cMyc* Human dermal fibroblasts	Neuronal rosette induction on PA6 stromal cells +Noggin and SB431542 6 days −Noggin and SB431542 8 days CD24+/CD184+/CD271−/CD44− cells selected by FACS Cultured in neural media (DMEM:F12+N2+B27+BDNF+GDNF+dcAMP) for 3 weeks w/o bFGF CD24+/CD184−/CD44− neurons selected by FACS	Neurons βIII-Tubulin+MAP2+	[101]
Retrovirus *OCT4*, *KLF4*, *SOX2*, *cMYC* Human dermal fibroblasts	Neuronal progenitor induction using dual-SMAD inhibition 9 days NB media 26 days–46 days	Neural progenitors 79% NESTIN+, small pop’n βIII-Tubulin+ Neurons Active Na^+^ channels K^+^ channels Produce action potentials 40% neurons Ca^2+^ spikes	[102]

Electrophysiological recordings also confirmed these cells to express tetrodotoxin sensitive voltage-activated currents and have active voltage-gated calcium channels. Together, this gave very convincing evidence for functional BFCNs with a relatively high purity. Israel and colleagues also performed extensive characterization of their neurons, including electrophysiological recordings. However, although 90% of the neurons were βIIITubulin^+^/MAP2^+^, the specific types of neurons produced remain unclear, with only 15% of neurons expressing VGluT1 and 8% expressing GABA. In the case of the cortical neurons generated by Shi and colleagues, these were found to include populations of both early and late born cortical neurons. These also formed functional synapses and expressed the glutamatergic marker PSD95 [100].

## 7. Modeling Impaired APP Processing from Patient-Specific Induced Pluripotent Stem Cells Reveals Considerable Variability

Varying AD pathologies were analyzed in these articles and all articles had in common an analysis of extracellular Aβ. Although some studies were unable to detect Aβ42 (as levels were below the detectable limits of the ELISA), it was striking to see how variable levels of Aβ40 were in the patient lines in comparison to the control/healthy cells. The familial lines carrying the APP duplication (APP^Dp^1/2) had increased Aβ40, although some sporadic lines (sAD1/2) reported no change in Aβ40 levels [96], and decreased Aβ40 was reported in at least three other familial lines carrying either a mutation in APP or PSEN1 [97,98]. Furthermore, increased Aβ42 was only reported in approximately one third of the patients [95,98,101,103]. Increased Aβ42:40 was noted in several familial PSEN1 and two familial APP(V717I) patient-derived neurons and it was apparent in at least two studies, that this elevation was due to increased Aβ42 [101,102,103]. The variation in observed secreted Aβ products may be dependent on the neuronal subtype analyzed, as elevated Aβ42 was observed in three of the five patients, where BFCNs were analyzed [95] and in both APP (V717I) patients, where forebrain neurons were produced [103]. In addition, increased Aβ42 was also observed in one AD patient, where cortical neurons were analyzed [98]. It was only the Down-syndrome-derived cortical neurons that displayed both increased secreted Aβ40 and Aβ42 levels [100]. In addition, in the same study, the increased Aβ42 was only detectable in neuron cultures that were older than 70 days and in APP (V717I)-derived neurons differentiated for 40–50 days, an increase in Aβ42 was also detectable [103]. Interestingly, in the case of PSEN1 (E280A) a screening of young pre-symptomatic carriers showed increased levels of Aβ42 in both plasma and CSF [105]. These studies therefore report a wide range of results for both Aβ40 and Aβ42 and may suggest that it could be necessary to have long-term culture protocols in order to see potentially relevant phenotypes. In the case of AD patients, we also know that a variation in expression levels of short Aβ peptides exists. For example, Aβ42 levels have been reported to be reduced in cerebral spinal fluid of patients compared to controls [106,107], whereas another study reported both increased and decreased Aβ42 in AD patients carrying PSEN mutations and decreased Aβ40 in the AD patient’s cerebral spinal fluids [108]. It was also interesting to see that the APP (V717I) iPS cell-derived neurons had an increase in Aβ38 [103]. Thus, it may be important for future studies on AD-derived iPS cells to perform long-term neuronal cultures and compare these directly to the Aβ levels in the original patient.

The evaluation for the presence of Aβ oligomers has been performed in only one study to date. Kondo and colleagues could detect the positive expression of the Aβ oligomer marker, NU1 and expression of the low weight oligomer marker, 11A1, in their cortical neurons, specifically localized as puncta throughout the neurons from both a familial APP and sporadic AD patient [98]. This was also the case for astrocytes generated from the same backgrounds. However, Aβ oligomers were not observed in another line, which had increased extracellular Aβ42:Aβ40. Postulation for this difference was made by the authors to support a hypothesis that AD may be classified as displaying either an extracellular or an intracellular phenotype.

APP processing was also studied in the AD iPS cell-derived cells by evaluating the effects that γ-secretase or β-secretase inhibitors had on the cultured cells. In general, most studies reported a decrease of Aβ40 or Aβ42 following treatment of the cells with a γ-secretase or β-secretase inhibitor [95,96,97,99,100,101]. One study also reported that a γ-secretase inhibitor decreased the production of APPsβ [103]. As an exception, two sporadic background iPS cell lines were reported to have increased Aβ40 levels following treatment with γ-secretase inhibitors [95]. The authors claimed that this may reflect the potential differences in APP processing in early onset disease* vs.* late onset disease as these two lines were derived from patients exhibiting early onset AD, or alternately, it may reflect patient-specific differences.

## 8. Tau Processing, Cell Death and Oxidative Stress in iPS Cell Lines Modeling AD

Levels of total and *p*-tau have been studied in only four of the reports to date. In one study, increased *p*-tau (Thr^231^) was reported in βIIITubulin^+^/MAP2^+ ^neurons in two familial AD-iPS carrying APP duplications and one sporadic AD-iPS cell line, however, this was not observed in a second sporadic AD-iPSC line when compared to control cells [96]. A second study has shown that Down-syndrome iPS cell-derived cortical neurons re-localized *p*-tau (Ser^202^ and Thr^205^) to the dendrites and cell bodies, which was not observed in the control cortical neurons, where diffuse staining was only observed in the axons [100]. Furthermore, this study showed that these neurons also secreted higher levels of total tau and *p*-tau (pSer^396^ and pThr^231^) over a 48 h period compared to the control neurons. Another study has reported both increased total tau and *p*-tau (Ser^262^), however the increase in pSer^262^ was only detectable in iPS cell-derived neurons differentiated for 100 days [103]. The final study revealed that no abnormal tau protein accumulation could be detected, or led to the production of tangles in two PSEN1-iPS cell neurons [99]. Interestingly, the tau pathology was noted in cells obtained from patients carrying mutations in the APP gene and not in the patient cells carrying PSEN1 mutations. However, given the limited numbers of studies analyzing *p*-tau, it may be difficult to conclude anything from this outcome. Again, it may be important that longer-term cultured cells are studied for such pathology as the latter study of *p*-tau on PSEN1-iPS was performed on neurons that were only 2 weeks old.

Cell death has only been reported in one study, namely in the Down-syndrome iPS cell-derived cortical neurons [100]. Cell death in the neurons was reportedly two-fold higher compared to the control neurons and was considered to be due to the secretion of tau into the medium. It was interesting to observe that despite some studies reporting increased levels of the toxic Aβ peptide Aβ42, this did not lead to increased cell death. In one study, a test on increased susceptibility to cell death by use of glutamate-induced excitation was performed on early sporadic AD iPS cell-derived BFCNs which had increased Aβ42 revealing that increased susceptibility could be seen, however this was also observed in a late sporadic AD iPS cell line which did not have elevated Aβ42 [95], meaning that the levels of increased Aβ42 alone could not be the primary reason for this susceptibility to cell death.

AD-iPS cell models may also be useful for studying oxidative stress. Whilst reports remain limited to date, one report showed that both familial and sporadic AD-iPS lines had increased levels of oxidative stress genes [98]. Elevated reactive oxygen species (ROS) was also detected both in the analyzed cortical neurons and astrocytes that were generated. Further research is clearly needed to further investigate the role of oxidative stress in these cell types compared to other AD* in vitro* cell models.

## 9. Hunting for New Genes of Interest in AD

It is apparent, that with current global gene/protein/lipid expression profiling technologies, human cells models of disease could be used to identify potentially new mechanisms. One recent study performed gene expression profiling (GEP) on immature neurons carrying PSEN1 FAD mutations and discovered several dysregulated genes [102]. Ten upregulated genes and four downregulated genes were validated and three upregulated genes, namely *NLRP2*,* ASB9* and *NDP* were investigated further by analyzing publically available GEPs performed on AD hippocampus and cDNA from the temporal pole of AD patients. *NLRP2*, a gene involved in inflammation was actually found to be downregulated in human AD temporal pole and no significant difference determined in the AD hippocampus. The gene *ASB9*, an ubiquitin ligase, was found to be upregulated in some AD patient temporal lobes, but not in the hippocampi. Finally, *NDP*, a gene thought to play an important role in CNS development was actually found to be significantly decreased in the AD patient hippocampi. Together, this study highlights that new genes can be discovered that could be used to pursue new mechanisms related to the disease, however, validation in the human brain is still an important and necessary measure to confirm the* in vitro* cell model findings.

## 10. Current Use of AD-Modeling Stem Cells for Compound Screening and Drug Testing

Only a rare cohort of studies has applied the use of stem cells derived from AD models to screen for novel compounds of interest or for testing recently identified drugs. These, to date, remain mostly restricted to mouse studies and primarily involve ESCs [109] although one study has used a non-AD human iPS cell model that is sensitive to Aβ aggregation for such purposes [110]. In one promising mouse study, ESCs were differentiated from a mouse model of AD (Tg2576) into an enriched population of pyramidal neurons and were subjected to a small molecule library to detect for inhibitors of Aβ40 [109]. Four candidate inhibitors were detected to induce over a 40% reduction in Aβ40 levels compared to controls, which included amiridine, icariin, phenelzine and progesterone. In the human study, healthy iPS cells were differentiated into forebrain neurons and subjected to an Aβ1-42 toxicity assay. These cells were then used to screen a GSK proprietary compound library for improvement in cell viability, which resulted in 19 hits, including a Cdk2 inhibitor. This field no doubt will grow in the coming years and will encompass AD-derived iPS cell lines which will help not only to discover new compounds of interest, but could also pave the way for patient-specific therapies.

## 11. Production of AD Isogenic Controls for Potential Gene/Cell Therapy

With the new revolution in gene editing, research has approached a new frontier for the generation of patient-specific cell therapies by correcting the patient’s diseased cells. This of course remains relevant for familial cases of AD and cases of known and diagnosed mutations. New techniques in genome-editing have been developed, which can be used to repair the particular disease causing mutation in a relatively simple manner by using transcription activator-like factor nucleases (TALENs), which are artificially produced restriction enzymes that specifically detect and bind to a desired nucleotide sequence in the genome and which initiate a double stranded break in the DNA. Homologous DNA fragments with the correct sequence need to be provided, so the cells can use these as a template to generate the correct sequence and thereby replacing the mutation. This method facilitates the generation of isogenic controls and control cell lines, which are absolutely identical to the patient iPS cells except for the repaired disease-causing mutation. Another such method is facilitated by clustered regularly interspaced short palindromic repeats (CRISPRs). This method is potentially faster and easier than the TALEN method. Correction of varying disease-related mutations in specific cell types has recently been performed and has even resulted in the correction of the disease in new progeny (in mice) when targeted in oocytes [111,112,113,114,115,116,117]. Some of these involve correction of frame-shifts [116], but it may also be possible to correct for single base pair mutations. One of the considered benefits of TALENs and CRISPRs is that there may be no residual ectopic sequences at the site of correction, although this depends on the strategy used for selecting for targeted clones, which may involve insertion of a selectable cassette. One potential drawback with this strategy is the potential to induce off-targeting genetic changes to other genomic sites that have either a similar or the same genetic sequence as that of the targeted sequence. The potential of targeting these other sites of course may lead to potential alteration in other genes throughout the genome. Such off-targeting has been observed in a handful of these studies [112,115,117], and therefore improvements in the design of the TALENs and CRISPRs for only the desired recognition target site may be needed before the next step to clinical transplantation is taken. To date, there is no literature on successful correction of an AD phenotype using either of these technologies; however, this area will no doubt be the focus of the next generation of research. Not only will it be important for the generation of healthy patient-specific cells that could be potentially transplanted, but corrected cell lines will form the ideal control cells needed for a more accurate interpretation of the AD phenotype in the diseased cells, due to the variation observed both between patients, but also between healthy age-matched controls.

## 12. Current General Limitations of Use of iPS Cells for Disease Modeling

There are several general limitations regarding the generation of iPS cells and differentiation into specific cellular subtypes, which are challenging and not very well understood. General limitations are caused by the limited understanding of the nature of iPS cells themselves and by their differentiation potential. In particular, the differentiation into a defined neural cell population is currently quite challenging. This is mainly because the developmentally relevant proteins and transcription factors, which are needed to mimic differentiation into a specific neural cell type, are not yet fully understood.

One general problem of using patient-specific iPS cells is the different epigenetic make-up and exposure to diverse environmental conditions every individual is facing. These differences have implications on comparative studies involving different patient-specific iPS cells, even between patients carrying the same pathological mutation. Despite these inter-patient differences, it has also been described that the reprogramming event itself can result in significant clone-to-clone variations, resulting in non-desired experimental background noise and even generation of non-disease related artifacts [118]. Currently, most studies involving the generation of patient-specific iPS cells involve the use of age- and gender-matched controls, which results in comparing epigenetically mismatched iPS cells. Isogenic controls generated via TALEN or CRISPR gene editing will be much more ideal for the study of disease-related cellular phenotypes and for pharmacological screens, which would help overcome this limitation.

Another general challenge is that only some of the aspects of a differentiated, aged cell can be restored to the state of pluripotency following reprogramming. Some of these may include an elongation of telomeres and restoration of functional mitochondria [119,120]. Other features pertaining to the original cell persist following reprogramming, such as acquired mutations, DNA damage, epigenetic changes and protein aggregation [121,122,123]. To date, it is still unclear what effect this has on the overall reprogramming efficiency and subsequent differentiation of iPS cells into the desired mature cell types. Moreover, iPS cells seem to retain an epigenetic memory, which makes them preferentially differentiate into their tissue of origin [124].

## 13. Hurdles Needed to be Overcome in Order to Recapitulate AD Faithfully in a Dish

One hurdle that needs to be overcome in order to accurately mimic the disease* in vitro* is the ability to produce the most relevant neurons for study. In order to develop* in vitro* models that can be used to screen for novel compounds for possible future treatments, it may be important to focus on areas of the brain that are affected earliest, and attempt to model the disease even before the symptoms first arise. One hope may be that iPS cells may be able to recapitulate earlier stages of the disease. Since Alzheimer’s disease pathology can first be detected in the entorhinal cortex, it might therefore be of interest to focus on differentiation of iPS cells into cell types affected in this area of the brain, namely pyramidal neurons with glutamate excitation and the varying GABAergic interneurons. It appears that it is the long projection neurons that are most vulnerable to developing pathology [18]. Short-axon projection cells such as spiny stellate cells apparently resist the pathology [18]. Short-axon local circuit cells also avoid pathology with the exception of the axo-axonic cells. It is particularly interesting that the vulnerable neurons are either un-myelinated or have only a thin sheath of myelin, as for e.g. heavily myelinated Betz cells and Meynert pyramidal cells also resist the pathology [18]. It may even be possible in the future to treat pre-symptomatic patients by large-scale screenings of the population. This would of course require earlier diagnostic tools for the disease for patients, which are still in general lacking, but of which several efforts are being undertaken [125,126,127,128]. In the case of the hippocampus, it is the CA1 neurons from the temporal medial lobe that are heavily affected by the disease, and ideally cross-comparisons with* in vitro* produced CA3 neurons, which are not affected as severely as the CA1 neurons would be ideal, however recapitulating these neurons* in vitro* is no easy task. In the CA1 region, which includes pyramidal neurons of at least three subtypes, there is also the supportive GABAergic interneuron population of which at least 20 different types are known [129]. Calretinin positive interneurons and somatostatin/parvalbumin positive interneurons (bistratified interneurons) in the CA1 are both affected in earlier stages of the disease [20]. In order to develop such protocols, a better understanding of the development of these neurons* in vivo* is required in order to mimic this process* ex vivo*. In the case of cortical pyramidal neurons, these are produced from progenitors located in the neocortical germinal zone in the dorsolateral wall of the telencephalon [130]. One recent report has shown the successful generation of cortical pyramidal neurons from both human ESC and iPS cells, which could successfully innervate the mouse brain [131], indicating there may be a strong future for developing efficient protocols for these cell types. In the case of the interneurons, these are generated in the ventral telencephalon and migrate to the neocortex [130]. Furthermore, although some markers can be used to distinguish pyramidal neurons from interneurons [129], additional markers of these neurons, in particularly, surface-specific markers are needed in order to improve selection and purification of these by use of FACS. Even though the brain regions affected by AD are composed of several neuronal subtypes as mentioned above, most differentiation protocols focus on the derivation of specific neural subtypes, which are mostly affected by the disease. These current protocols achieve in some cases good enrichment of a certain neural subpopulation (see Table 2). Despite the varying outcomes of different protocols there is also the problem of different results from the different iPS cell clones from the same patient cell line. Furthermore, neural differentiation is a complex scenario, which is dependent on internal and external morphogenic cues, gene expression and transcription factor activity in a spatio-temporal manner [132].

Another significant hurdle is overcoming the lack of knowledge of the types of cells that are currently being used for analyses. Heterogeneity itself may not be a problem, if we can re-create the same heterogeneity observed in the specific regions of the brain affected. However, several different approaches and protocols currently exist for differentiation of ESC and iPS cells into cortical neurons [100], BFCNs [95], other neurons like dopaminergic neurons mostly affected in Parkinson’s disease [133] and astrocytes [134,135]. Some of these differentiation protocols may show variation in differentiation efficiency between cell lines, but even from experiment to experiment using the same clone but at different time points [136].

Many of the differentiation protocols in the AD iPS cell papers to date have produced neurons which are βIIITubulin^+^ and MAP2^+^ [95,96,97,99,101] using neuronal induction factors, such as, BDNF, GDNF, N2 and B27 (see Table 2). Although it is promising to see phenotypic hallmarks of AD recapitulated at a cellular level using these differentiation protocols, there remain variations in the phenotypes created. This may be due to the differences in timing of differentiation, some degree of cell heterogeneity and the lack of clear understanding of the types of neurons generated. It therefore remains difficult to make a direct comparison of the conducted approaches and analyses of AD iPS cells. For example, it might well be possible that the observed elevated expression of stress-related genes and ROS as well as the formation of Aβ oligomers found in the SATB2^+^ and TBR1^+^ neurons is only observable in this specific sub-population and not detectable in other βIIITubulin^+^ and MAP2^+^ neurons.

In conclusion, there are many differences amongst the final neural cell population generated by the differentiation protocols, as well as in the final composition of neural subtypes generated. The reproducible detection of an AD related phenotype is very much dependent on the generation of predictable and fully matured brain region-specific neurons. Therefore, it would be relevant to combine the phenotypic observations so far gathered and routinely check all AD iPS cell models for the presence or absence of all of these disease hallmarks.

Even though tremendous advances have been made in the generation of AD iPS cells and subsequent differentiation into cortical neurons, other neurons and glia, the analysis of the cellular disease phenotype is still the most challenging aspect of this cellular model of AD. One of the most profound problems is the lack of reliable reproducibility of the differentiation protocols and the clonal variation even amongst iPS cell clones from the same patient, which could be responsible for the varying outcomes in the results. One explanation could be the incomplete reset of the cellular epigenetic landscape to the pluripotent state and current limitations of differentiation protocols, which fail to produce functional and specific neuronal subtypes. This could possibly be contributing to the observed lack of a disease phenotype and also the diversity in the observed disease phenotypes. One of the sporadic AD iPS cell lines showed no phenotype whatsoever, which could mean that there are either unknown phenotypic hallmarks related to AD, or simply, in this case, the neurons were not matured enough to display a disease phenotype. The lack of mature neuronal differentiation is supported by the findings that another APP related mutation (APPV717I) showed a marked increase in Aβ42 levels, which increased during the course of the differentiation protocol. A comparative analysis of APP and APP cleavage products starting from day 9 until day 100 clearly showed that a significant increase of Aβ42 was not detectable before day 40 during the terminal differentiation protocol [103]. These two approaches underline the necessity of optimized differentiation protocols in terms of duration and timepoint of analyses of the disease phenotype. Another plausible explanation for the absence of a cellular SAD phenotype could be due to altered Aβ clearance in the patients. It has been shown by several groups that astrocytes may contribute to the Aβ clearance by restricting the inflammatory response in the brain [137,138]. Interestingly, APOE4, which is a risk factor for SAD, is expressed in astrocytes implying an important functional role of these cells in the neurodegenerative progression in the patients [139]. Microglia certainly also play an important role in Aβ clearance [140]. These cell types and their impact in AD* in vitro* systems remain largely unexplored. Nevertheless, it was also possible to observe an increase in phosphorylation of tau (Thr^231^) and an increase in GSK3 beta activity, in the two APP duplication iPS cell models and in one of the sporadic AD iPS cell models [96]. The use of more defined neural subtypes could be more beneficial in dissecting the underlying causes of AD progression. These are also encouraging findings for validation in using these cellular models to identify cellular changes in AD. Different groups have reported the production of AD iPS cells and used different approaches to perform neural differentiation and analyses of these cells. This makes it difficult to establish a common cellular phenotype to set as a baseline for AD iPS cell models. However, this is very necessary in order to ensure that a lack of phenotype or a novel phenotype is not caused by insufficient neural subtype differentiation.

Overcoming the variation in the AD pathologies of analyzed AD-iPS-derived neural cells is important. Whether this variation is reflective of patient variability or cell line variability remains unclear. However, one way to overcome this problem would be to make sure at least three clones of each patient are produced, and that these produced identical phenotypes. It may also be important to have patient medical history that can verify pathology observed when first analyzing results. It is evident that not all iPS cell clones recapitulate results, and those that do not should be discarded or eliminated from analyses and interpretation. It is also clear that our lack of understanding of the cell types that are being analyzed could impede dramatically on our results and interpretations of them. This is a difficult task to overcome, as the complexity of the brain is colossal. It requires significant years of basic research into identifying the development cues of the neurons and neural cells of interest for the disease. Although, a recent promising study has revealed that 3D brain structures can be recreated* in vitro* from differentiated human embryonic stem cells [141], revealing that it may be possible to even recreate specific sub-regions of the brain within a dish in the future.

## 14. Future Induction of an AD Phenotype Using Components that Introduce Cellular Stress

Issues in resetting the biological clock during reprogramming could quite possibly explain the difficulties observed in obtaining ideal phenotypes in some AD iPS cell models. This does not come as a surprise since AD is a disease in which not only the malfunction of AD related genes, but also the aging of cells, as well as the whole organism is involved. This potentially makes the fundamental use of* in vitro* AD iPS cell systems questionable. However, some research groups have started to use cellular stresses to provoke accelerated aging in* in vitro* produced neural cells and have even introduced systems, which overexpress genes related to premature aging. This could lead to the development of shorter differentiation protocols, which would be of extreme benefit both for researchers and for the eventual benefits for patients.

In one study, it was possible to alleviate Aβ oligomer-induced cellular stress using docosahexaenoic acid (DHA) in neurons derived from iPS cells [98]. Since it is known that oxidative stress is a key hallmark of Alzheimer’s disease and accelerates the diseases progression [142], ROS could be useful in triggering a disease phenotype in SAD iPS cell-derived neurons where no disease phenotype is observable or to accelerate the cellular disease and aging process itself. Mitochondria generate energy via oxidative phosphorylation and ROS is the byproduct for this energy generation. This observation led to the free radical hypothesis of ageing, which makes ROS species responsible for accumulative cellular damage over lifetime [143]. Currently, aerobic metabolism and the corresponding generation of ROS is still the most widely accepted cause of ageing, but little is known about the intracellular targets of ROS and how oxygen manipulation of these influences lifespan [144]. Another widely used ROS species is hydrogen peroxide (H_2_O_2_), which belongs to the exogenous ROS species, causing mostly DNA damage and which induced an apoptotic cellular response at high doses. The usage of this kind of stressor has therefore a lasting effect due to the DNA damage; however, it is not clear if these mutations directly cause a phenotype. On the other hand, a recent study showed that exposure of rat NPCs to H_2_O_2_ may actually be beneficial and induce neurogenesis [145], which is in contrast to the proposed damaging effect H_2_O_2_. This report also showed that low dosages of H_2_O_2_ induced proliferation of rat NPC cells, and even modified their differentiation potential towards an oligodendrocyte fate. This is a particularly interesting aspect since inflammation processes in the brain caused by H_2_O_2_ have been reported [146]. Unfortunately, the preferred differentiated neural subtype by low dosage exposure to H_2_O_2_ are oligodendrocytes, which would not be useful in replacing the degenerated pyramidal, cortical or cholinergic neurons, which are mostly affected by neurodegeneration in AD. Another technique, which is widely used to stress cells, includes serum starvation (which in the case of neural cells involves withdrawal of B27). This has been shown to robustly induce autophagy and neural death [147] and therefore a reduction of B27 in the neural media could be used to mimic stress and induce autophagy. Currently, none of the AD iPS cell published studies have used ROS species or serum starvation to provoke a more profound disease phenotype.

## 15. Induction of an AD Phenotype by Manipulating the Gene Expression of Age Inducing genes

Another possible approach to mimic ageing in a dish could be to activate or repress key regulatory genes involved in the ageing process. A recent report revealed that overexpression of progerin (which when occurs in humans, causes Hutchinson-Gilford progeria) in an iPS cell model of Parkinson’s disease resulted in an accelerated aged phenotype [148]. This cell model revealed pronounced dendritic degeneration, progressive loss of tyrosine hydroxylase expression, enlarged mitochondria and Lewy-body-precursor inclusions, which are indicative to the fact that the induced ageing was successful.

Other strategies to induce accelerated aging could involve RNAi-mediated knockdown of relevant targets such as sirtuin 1 (SIRT1), repressor element 1-silencing transcription factor (REST) or vacuolar protein sorting 41 (VPS41). Further genes of interest have been identified in *C. elegans*, which are also involved in autophagy lysosomal trafficking and shown to convey neuroprotective features. Amongst these are autophagy related 7 (ATG7) and PDZ domain containing family, member 1 (GIPC) [149]. SIRT1 has been shown to be involved in healthy ageing and longevity [150,151] and appears to be neuroprotective in AD [152]. Moreover, REST induces the expression of stress response genes and is neuroprotective [153]. VPS41 is involved in lysosomal trafficking and overexpression of this protein has been shown to enhance clearance of misfolded alpha synuclein [154]. A knockdown of VPS41 appears to hinder the lysosomal complex function and formation and accelerate accumulation of toxic misfolded proteins including Aβ. In particular, genes involved in autophagy could be of interest since the clearing of misfolded Aβ is believed to occur via autophagy and a downregulation or ablation of genes in this pathway could induce ageing as well as enhance the AD phenotype related to autophagy. Other studies have implied an important role for Beclin1 in autophagy and even APP processing [155], which makes this gene an interesting target as well. Clearly a systematic knockdown approach via RNAi targeting components of the autophagy and lysosomal pathways would be an amenable approach for identifying suitable targets that could induce ageing and accelerate the cellular pathology of AD iPS cell-derived neurons.

## 16. Conclusions and Future Perspectives

There are obviously still some hurdles that need to be overcome before science can faithfully recapitulate AD in a dish using iPS cells that might provide benefit to AD patients. It also remains to be seen if cell therapy by transplantation of AD-corrected iPS-derived neural cells could be of benefit to patients, by assessing integration of grafts into the brain and/or other related effects such as inflammation, or if AD-iPS derived disease models could help to deliver new and more advanced therapies to the patients. It is clear from the studies performed so far that more research is required. In keeping perspective, this research is aimed for the development of new and better medicines that can treat the disease long-term, rather than medicines that apply temporary brakes on it, and ultimately we are searching for a cure, which may totally alleviate the disease. The benefits to the community both at a societal level, but also at an economical level, are tremendous and would positively benefit millions of people around the globe.

What is evident, however, is that there is a clear step towards translational medicine for pluripotent stem cells, and in particular for treatment of disease. This is most striking in the case of other neurodegenerative disease such as Parkinson’s disease [156,157]. Ultimately, human iPS cells will help to contribute detailed knowledge on AD mechanisms and might even lead to breakthroughs that could allow clinicians to develop earlier diagnoses, or be used for patient individualized medication and potentially for future cell transplantations. In considering how far we have come with the advancement of iPS technologies, and in the few years since the implementation of the technology, it is likely that the path ahead will unveil potentially significant advances in the treatment of the disease.

## Abbreviations

B27: (B27 supplement)
BDNF: (brain-derived neurotrophic factor)
bFGF: (basic fibroblast growth factor)
cAMP: (cyclic AMP)
dcAMP: (dibutryl cyclic AMP)
EB: (embryoid body)
EGF: (epidermal growth factor)
EGFP: (enhanced green fluorescent protein)
FACS: (fluorescence activated cell sorting)
GDNF: (glial cell-derived neurotrophic factor)
IGF: (insulin growth factor)
IPS cell: (induced pluripotent stem cells)
It-NES: (neuroepithelial stem cells)
KSR: (knockout serum replacement)
N2: (N2 supplement)
NB: (neural basal media)
NGF: (nerve growth factor)
NPC: (neural progenitor cell)
RA: (retinoic acid)
seq.: (sequencing)
SHH: (sonic hedgehog)
wks: (weeks)
w/o: (without)
3N: (modified bold 3N medium)
